# Size-inclusive advertising in the Asian fashion market: Female consumers’ responses to a plus-size vs. Thin-size model in South Korea

**DOI:** 10.1371/journal.pone.0304989

**Published:** 2024-06-17

**Authors:** Sunwoo Kim, Su Jin Yang

**Affiliations:** 1 Research Institute of Human Ecology, Seoul National University, Seoul, South Korea; 2 Department of Consumer Science & Living Culture Industry, Sungshin Women’s University, Seoul, South Korea; Sri Sivasubramaniya Nadar College of Engineering, INDIA

## Abstract

Global fashion brands have embraced size-inclusive advertising featuring plus-size models, yet the responses of Asian consumers to such advertising—where the average body size of women is smaller than in Western markets—have garnered little attention. This study, utilizing the S-O-R model, aimed to investigate whether the relationships among perceived actual and ideal self-congruence, perceived attractiveness and familiarity of a fashion model, and purchase intention vary based on the body size of the fashion model. We tested the hypothesized relationships using ANCOVA, confirmatory factor analysis, and multi-group structural equation modeling, analyzing 623 online survey responses from South Korean female consumers. Actual self-congruence had a greater influence on perceived familiarity in consumers exposed to a thin-sized model compared to those exposed to a plus-sized model. In contrast, ideal self-congruence had a more significant positive impact on the perceived physical attractiveness of the plus-size model than the thin-size model. Furthermore, the plus-size model’s perceived physical attractiveness had a more significant positive effect on purchase intention than that of the thin-size model. This study highlights the importance of crafting advertising images that portray plus-size models as physically attractive to elicit favorable responses from Asian consumers.

## Introduction

Contemporary fashion advertising frequently perpetuates distorted beauty standards by predominantly featuring models with unrealistically thin physiques, leading to considerable criticism [1, 2]. This portrayal impacts consumers’ self-image as they often compare their own body sizes with those of fashion models, resulting in dissatisfaction with their average-sized bodies [3]. The widespread adherence to this norm might marginalize consumers who do not conform to the desired thin bodies, making them feel excluded from the fashion narrative and potentially identifying them as part of a neglected minority group [3]. More concerning is the potential for indiscriminate exposure to these beauty ideals to precipitate significant health issues, including eating disorders and a higher likelihood of undergoing cosmetic surgery [1, 3]. In response to these sociopsychological concerns, numerous fashion brands have begun to incorporate plus-size models in their advertising campaigns, conveying the message that “everyone’s body is beautiful as it is” [4]. Consequently, the representation of plus-size models, typically US women’s apparel sizes 14 and above, gained traction in Western markets around the early 2010s [4, 5]. The shift towards size inclusivity more accurately reflects the average Western female body, in contrast to the sizes 0 to 2 commonly associated with conventional fashion models, especially considering that the average body size of American women falls between sizes 16 and 18 [6, 7].

Numerous studies have explored the rise of size-inclusive advertising as a predominant strategy, focusing on its sociopsychological benefits, such as increased body satisfaction, positive self-image, and improved psychological well-being among consumers [8, 9]. However, the literature on the economic benefits of size-inclusive advertising has presented a lack of consistency. Some research has indicated that the use of plus-size models in fashion advertising does not invariably influence consumers’ purchasing behavior towards the promoted items [10, 11]. The traditional approach of fashion advertising, which focuses on creating compelling brand personas that resonate with consumers’ desirable aspirations and ideals, may be responsible for this phenomenon, and this approach has been established and perpetuated over many years. Nevertheless, recent studies have found that evolving market dynamics, particularly among young customers, are mitigating the economic drawbacks associated with including plus-size models [4].

In contrast to Western fashion markets, there is scant scholarly research on Asian consumers’ reactions to plus-sized models. Understanding Asian consumers’ responses to plus-size models is imperative because the standards for such models are rooted in Western concepts and promoted by major global fashion brands based in Western markets. In 2022, World Obesity reported obesity rates among adult women across 200 countries, labeling a body mass index (BMI) exceeding 30 as obesity [12]. The data revealed significant disparities in obesity rates across national markets. Specifically, the US ranked 36th with a 43.82% obesity rate, while China and South Korea, representing East Asia, ranked 190st and 194th, with obesity rates of 7.78% and 5.65%, respectively [12]. This difference in obesity rates suggests that Western consumers, with a closer body size in line with plus-size models, might be more accepting of such models. Conversely, Asian consumers may be less receptive to larger models that diverge from their actual body sizes. The contrast in average BMI between adult women in the US and SouthKorea further supports this assertion, with the US average at 26.5 and South Korea at 22.6 [13].

The differing responses to plus-size models in Asian versus Western markets could be due to the impact of body image perceptions on the psychological well-being of Eastern women. Awareness of body positivity in Eastern cultures, especially among older generations, has been comparatively lower than in the West [14]. Moreover, among younger generations in South Korea, weight control through dieting often takes precedence over body positivity [5]. Thus, exploring South Korean consumers’ responses to size-inclusive advertising featuring plus-size models that conform to Western-defined body size standards is imperative. South Korean consumers were chosen as the focus due to their significant cultural and fashion influence globally [15, 16] and their exceptional adaptability to emerging trends [17], suggesting that plus-size models might gain popularity more swiftly in South Korea than in other Asian countries.

Accordingly, the present study investigates the effect of size-inclusive advertising featuring a plus-size model on the purchase intention of South Korean consumers compared to conventional advertising with a thin-size model. We developed a research framework based on the stimuli-organism-response (S-O-R) model [18], presenting two advertising materials as stimuli: one featuring a size-inclusive (i.e., plus-size) fashion apparel model, and the other featuring a conventional (i.e., thin-size) one. Additionally, organism variables such as self-congruence (i.e., actual self-congruence and ideal self-congruence) [10, 19] as well as the two types of perceived attractiveness (i.e., perceived physical attractiveness and perceived familiarity) [20–23] were included to assess their influence on consumers’ intention to purchase products advertised by the two types of models.

The findings are expected to enrich the literature on models and advertising by elucidating the distinct roles of self-congruence and perceived attractiveness in shaping purchase intention based on fashion models’ body sizes. Notably, South Korean female consumers, despite their generally slender physiques, have exhibited increasing interest in plus-size yet attractive fashion models. Their preference for attractive plus-size models may be intensified when perceiving ideal self-congruence. These insights often provide valuable perspectives on incorporating plus-size fashion models in South Korea and potentially across the broader East Asian fashion industry.

## Literature review and hypothesis development

### The S-O-R model

The S-O-R model, developed by Mehrabian and Russell, elucidates the process by which human beings respond to stimuli in three steps: stimuli-organism-response [18]. Given its significant focus on consumers’ responses to new commercials and marketing campaigns, the S-O-R model has become one of the most utilized frameworks in advertising research, adopting advertising elements as stimuli [24, 25]. Specifically, environmental stimuli that influence consumer decision-making fall under the category of stimuli, which encompasses various marketing aspects (i.e., advertising, brand, pricing, and distribution channels) [24]. The organism component refers to consumers’ feelings, emotions, and perceptions in response to these stimuli [25], while the response denotes individuals’ specific reactions towards marketing materials, such as either approaching or avoiding them [24].

**Fig 1:**
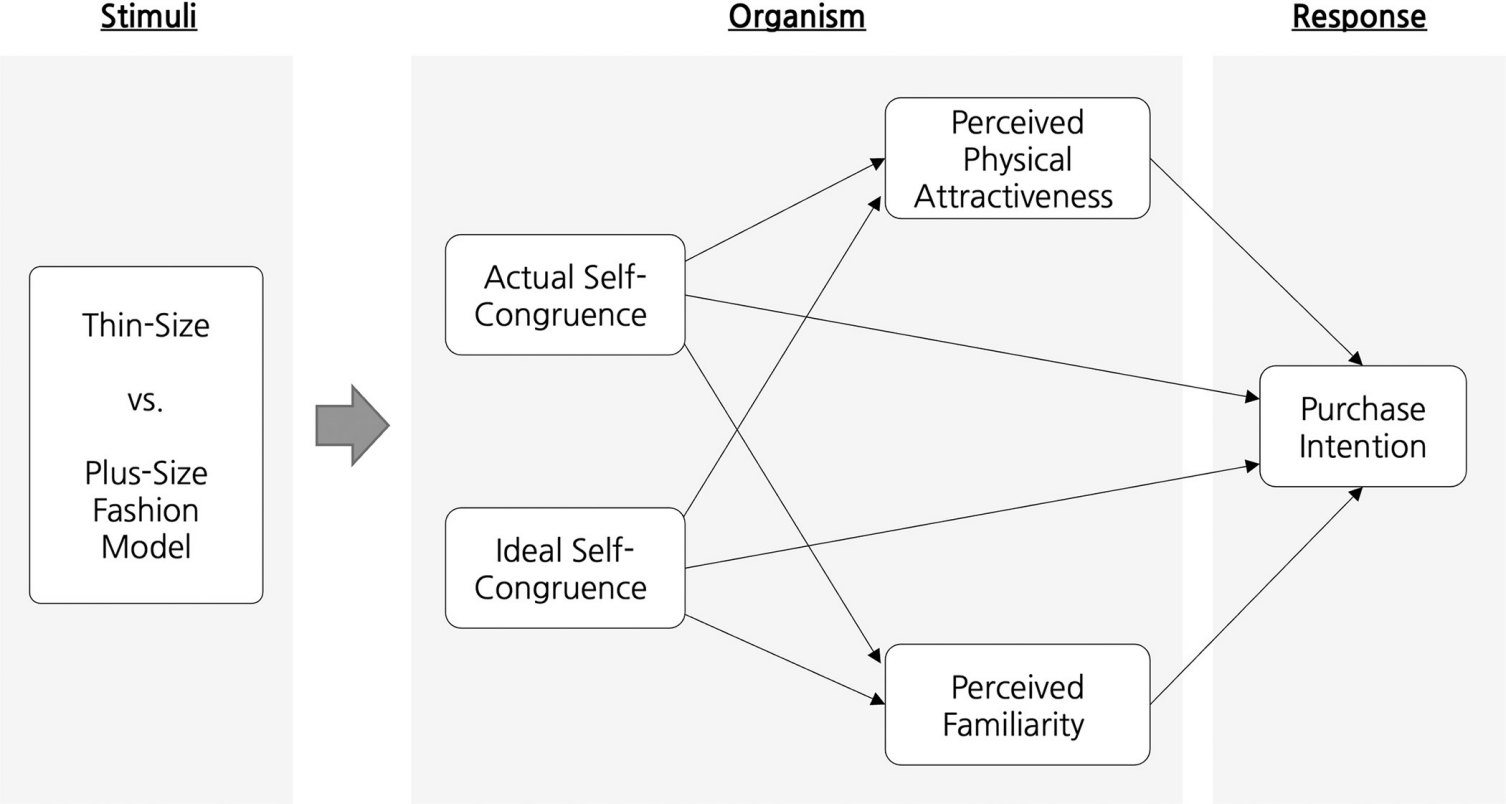
Conceptual framework based on the S-O-R model.

In the advertising domain, the attributes of endorsers represent some of the most influential stimuli, determining advertising outcomes from both sociopsychological and economic perspectives [26–28]. Notably, the body size of endorsers has emerged as a significant attribute [4]. Therefore, this study presented two types of stimuli to understand consumers’ responses: a plus-size model as a new advertising stimulus and a thin-size model as a conventional stimulus. Hwang and Shin noted that product descriptions in online retail environments often emphasize what fashion models are wearing rather than the products themselves [29]. Moreover, the attributes of advertising models can affect consumers’ responses, including their purchase intention for the advertised products [30]. The results of Hwang and Shin’s study highlighted a crucial link between the body sizes of fashion models and their influence on consumers’ intentions to purchase the advertised products [29]. Furthermore, based on congruence theory [31, 32], this study identified two layers of organism variables: self-congruence with advertising models and perceived attractiveness. Consumers generally align their expenditures with their self-concept [33] and the perceived congruence between the attractiveness of advertising models and consumers’ self-concept influences their responses to the advertising [34]. As depicted in Fig 1, consumers’ self-congruence with an advertising model encompasses not only whether it corresponds to their actual selves but also whether it conforms to their ideal selves [31, 32]. Studies have also shown that consumers’ perceived physical attractiveness and perceived familiarity of an advertising model elicit favorable responses from consumers [23, 26, 28, 35] and ultimately enhance purchase intention [36]. Accordingly, a research framework, as illustrated in Fig 1, was developed.

### Self-congruence and fashion models’ attractiveness

Consumers cultivate and uphold their self-concept, which is a comprehensive construct that encompasses their abilities, individuality, appearance, and personality, through their consumption behaviors [32]. Among various self-concept dimensions, prior research has primarily focused on two sorts of self-concept: the actual self, which reflects individuals’ perceptions of themselves in reality, and the ideal self, which represents how individuals ideally perceive themselves [37]. Empirical studies have shown that when consumers perceive congruence between themselves and a model in an advertisement, it positively affects their preferences, increases their purchase intentions, and enhances the effectiveness of the advertisement [33, 37–39].

Previous studies applying self-congruity theory to fashion advertising models have demonstrated that consumers’ purchase intentions for advertised fashion products increase when they perceive congruence between their ideal or actual selves and the advertising model [10, 19]. Given the scarcity of empirical evidence on inclusive advertisements [40], it is imperative to prioritize the examination of purchase intention as a key determinant of its effectiveness in the digital marketing realm [41]. Thus, this study empirically tests hypotheses related to the influence of perceived actual and ideal self-congruence with both thin and plus-size models on purchase intentions for advertised apparel products. Accordingly, we developed the following hypothesis:

H1: (a) Actual self-congruence and (b) ideal self-congruence have a positive influence on purchase intention.

Attractiveness refers to an indescribable psychological attraction that captivates and arouses interest, enhancing the persuasive efficacy of advertising models [42]. As an intuitive and immediately noticeable trait, consumers quickly discern the attractiveness of advertising models [43]. Considering attractiveness as a multidimensional concept [26–28], this study concentrates on physical attractiveness and familiarity as its sub-dimensions. Erdogan’s study on the impact of source characteristics on persuasion communication found that physical attractiveness and familiarity serve as crucial sub-dimensions in shaping the perception of advertising models as attractive [26].

The physical attractiveness of advertising models has been extensively explored in the advertising literature [26, 28, 35]. Since people subconsciously accept the proposition that “Beauty is good,” a fashion model’s physical attractiveness produces a halo effect, which enhances the attraction of the advertised products [20]. A related concept, familiarity, refers to the degree to which consumers feel close to and comfortable with an advertising model [22]. Individuals form perceived familiarity as they accumulate direct and indirect consumption experiences [21]. In modern society, when advertisements are bombarded, consumers prefer familiar models since such models require less cognitive effort to process the stimuli, resulting in more favorable consequences than unfamiliar models [22, 23]. Given the proliferation of new media channels (e.g., social media platforms), consumers’ perceived familiarity with fashion models has become crucial to commercial success [44]. Thus, we present our second hypothesis:

H2: (a) Perceived physical attractiveness and (b) perceived familiarity of a fashion apparel model have a positive influence on purchase intention.

Being in congruence with advertising sources is widely acknowledged as a reliable indicator of having a positive perception of the sources’ attributes [45]. More precisely, when consumers perceive higher congruence between their self-concept and advertising endorsers, they are more likely to evaluate the endorsers’ attributes favorably [45]. Prior research has concentrated on diverse attributes, including perceived credibility, personality traits, and expertise of advertising endorsers [46–48]. Perceived attractiveness has also been examined in several studies [45]. Therefore, we predict dual-path relationships between the two types of congruence, namely ideal self-congruence and actual self-congruence, as well as two dimensions of perceived attractiveness, namely physical attractiveness and familiarity:

H3: Actual self-congruence has a positive influence on (a) perceived physical attractiveness and (b) perceived familiarity of a fashion apparel model.
H4: Ideal self-congruence has a positive influence on (a) perceived physical attractiveness and (b) perceived familiarity of a fashion apparel model.

### South Korean female consumers’ perceptions of plus- vs. thin-size models

The long-standing preference for extremely thin bodies has hindered the acceptance of size-inclusive advertising in the South Korean fashion market [5]. According to a report by a South Korean government agency [49], among women aged 10 to 39 in South Korea, around 50% of the population falls into the BMI range of 18.5 to 23.0, which is within the average range. The prevalence rate of obesity, defined as a BMI over 25, is a mere 17% for those in their 20s and 25.7% for those in their 30s. Among those aged 60 and above, the prevalence rate of obesity increases to 40%. However, the issue of underweight teenagers is more concerning due to its significant rise from 21% in 2015 to 34.6% in 2021. Moreover, South Korean apparel brands targeting female consumers typically offer limited sizes from 44 (24-inch waist) to 66 (28-inch waist), whereas their US counterparts provide a broader range of sizes from 00 (22.5-inch waist) to 24 (44-inch waist). Out of all female fashion manufacturers in South Korea, only 1.6% have produced 88-size (32-inch waist) apparel products, which is the largest size in the South Korean fashion industry [50]. Similarly, the South Korean fashion industry has yet to establish standardized size ranges for plus-size consumers [51]. Hence, the fashion market catering to plus-size consumers in South Korea requires special attention.

Given the distinct sizing system in the South Korean fashion industry, we expected differences in key variables in this study according to the body sizes of fashion models. With respect to self-congruence, thin-size models, aligning more closely with South Korean women’s preferences and being stereotypically favored, are expected to result in more favorable advertising outcomes [52]. In South Korea, where the average female body size approximates that of a thin-size US model (US size 4) [51], we predicted that perceived actual self-congruence would be greater when exposed to a thin-size model compared to a plus-size model. Additionally, the prevalent pursuit of thinness ideals in South Korea [53] may lead females to perceive a thin-size model as more congruent with their ideal selves compared to a plus-size model.

Skepticism towards size-inclusive advertising has become a common reaction in South Korea, as fashion companies in the country began to adopt this approach approximately a decade later than their Western counterparts [54]. A prior study analyzing internet hashtags in English and Korean for “#plussizemodel” found that the Korean hashtag was predominantly associated with negative advertising implications, whereas the English hashtag was linked to body appreciation, improved body image, and psychological well-being [5]. These findings imply that South Koreans continue to favor thin-size models, indicating a lack of size inclusivity in the country [5]. Therefore, South Korean consumers may perceive a thin-size model as more physically attractive and familiar than a plus-size model. Furthermore, we predicted that a thin-size advertising model would elicit higher purchase intentions for the promoted apparel products compared to a plus-size model, leading to the next hypothesis:

H5: Consumers exposed to a thin-size model perceive significantly higher levels of (a) actual self-congruence, (b) ideal self-congruence, (c) physical attractiveness, (d) familiarity, and (e) purchase intention than those exposed to a plus-size model.

### Moderating effects of fashion models’ body sizes

This study examined how South Korean consumers form purchase intentions toward fashion products advertised by a plus-size model, incorporating the body size of fashion models as a moderating factor to investigate its influence on the hypothesized relationships. Given the cultural preference for a slender body shape resembling that of a thin-size model, South Korean females might favor such a physique due to societal expectations to maintain slimness, often perceived as indicative of motivation to exercise and maintain appearance [55]. Moreover, South Korean females possess heightened self-awareness and are discerning regarding their physical appearance, particularly their body shape [56]. Therefore, if a South Korean female believes her self-concept is congruent with a fashion model in an advertisement, and this congruence facilitates her purchase intention of the advertised fashion products, we anticipated a stronger influence from a thin-size model compared to a plus-size model in terms of both their perceived actual and ideal self-congruence.

We also predicted that perceived physical attractiveness and familiarity would have a stronger influence on purchase intention when exposed to a thin-size model as opposed to a plus-size model. In terms of perceived physical attractiveness, South Korean culture has long adhered to beauty standards that prioritize a slender body size for decades [53]. More specifically, we anticipated that the persuasive communication effect of perceived physical attractiveness, considering a thin-size model as a source characteristic in advertising, would be more substantial than that associated with a plus-size model [26]. In terms of perceived familiarity as a source characteristic, we predicted that the influence of perceived familiarity with a thin-size model would outweigh that associated with a plus-size model. The premise of this prediction is that South Korean consumers’ limited exposure to plus-size models has led to lower perceived familiarity, potentially leading to a less pronounced influence in purchase intention [5].

H6: The influences of (a) actual self-congruence and (b) ideal self-congruence on purchase intention are greater when exposed to a thin-size model than when exposed to a plus-size model.
H7: The influences of (a) perceived physical attractiveness and (b) perceived familiarity on purchase intention are greater when exposed to a thin-size model than when exposed to a plus-size model.

Perceived self-congruence when exposed to advertising models is widely acknowledged as an antecedent influencing the perception of attributes related to the models, such as perceived physical attractiveness and perceived familiarity [22, 45]. Additionally, South Korean consumers have long contended with social pressure to conform to a thin body ideal, internalizing this body shape as their ideal standard of beauty [53]. Consequently, we posit that the influence of perceived self-congruence, encompassing both actual and ideal selves, on perceived physical attractiveness would be more pronounced when consumers are presented with a thin-size model rather than a plus-size model. Considering the limited range of apparel sizes available in South Korea [54] and the nation’s notably low average BMI compared to other OECD countries [12], we anticipated that the influence of perceived self-congruence, encompassing both actual and ideal selves, on perceived familiarity would be stronger when consumers are exposed to a thin-size model compared to a plus-size model.

H8: The influences of (a) actual self-congruence and (b) ideal self-congruence on perceived physical attractiveness are greater when exposed to a thin-size model than when exposed to a plus-size model.
H9: The influences of (a) actual self-congruence and (b) ideal self-congruence on perceived familiarity are greater when exposed to a thin-size model than when exposed to a plus-size model.

## Methods

### Stimuli development

We created advertising stimuli in a format commonly used in online fashion distribution channels, as size-inclusive advertisements in South Korea are predominantly distributed through those channels [5]. Initially, we acquired an image of a female fashion model from a stock image database, ensuring adherence to ethical and legal guidelines. The acquired image portrays a Caucasian woman, reflecting the common practice of featuring Caucasian plus-size models in the advertising of primarily global fashion brands rather than local fashion brands in the South Korean market [14]. To mitigate the potential influence of clothing design, the model wore a basic outfit consisting of a plain white T-shirt and blue jeans [57]. Furthermore, the image’s background was devoid of any brand names to minimize potential effects associated with brand awareness, reputation, and preference. However, English advertising phrases were added to ensure that the research participants would clearly recognize the stimuli as an advertisement for a global fashion brand.

Using the acquired image, we developed two contrasting research stimuli, featuring a thin-size model and a plus-size model, based on Korean females’ perceptions of the models’ body sizes. Initially, we modified the acquired woman’s image using Photoshop to create seven size levels, ranging from “*very thin-size*” to “*very plus-size*” body sizes, thus diversifying the original image into seven modified versions representing different body sizes as preliminary stimuli. These images were used in a pre-test with South Korean females (*N* = 108; *M*_age_ = 31.065, *SD*_age_ = 5.100), who were asked to rate the body sizes of the seven images using a figure rating scale of body silhouettes consisting of nine levels representing different body sizes (1 = “extremely thin size” and 9 = “extremely plus size”), as proposed in Bays et al.’s study [57]. This scale was developed to measure subjective perceptions of body image alongside nine-level BMI ranges [58]. The scale allowed us to understand how South Korean female consumers perceived the seven modified images’ BMI levels. Based on these results, we selected two contrasting model images [58]: the thin-size model (*M*_thin-size_ = 2.352, *SD*_thin-size_ = 0.801, BMI = 20.5 to 22.3) and the plus-size model (*M*_plus-size_ = 6.815, *SD*_plus-size_ = 1.024, BMI = 32.5 to 36.5). A t-test confirmed a statistically significant difference in perceived body sizes between the two stimuli (*t*(107) = 37.595, *p* < .001).

### Sample, design, and procedures

This study surveyed 623 South Korean women in their 20s and 30s (*M*_age_ = 31.369, *SD*_age_ = 4.981). Participants were divided into two groups: one exposed to a thin-size model stimulus (*n* = 316, *M*_age_ = 31.316, *SD*_age_ = 5.024), and the other to a plus-size model stimulus (*n* = 307, *M*_age_ = 31.424, *SD*_age_ = 4.944; *t*(621) = -0.837, *p* >.05). We chose this relatively young age group because, in South Korea, the promotion of body inclusivity has mostly targeted this particular consumer segment [14]. The average BMIs of the two stimulus groups did not differ statistically, indicating comparable mean BMI values between the two groups (the thin-size model group: *M*_BMI_ = 21.541, *SD*_BMI_ = 3.340; the plus-size model group: *M*_BMI_ = 21.776, *SD*_BMI_ = 3.669; *t*(621) = -0.256, *p* > .05). The Institutional Review Board of Seoul National University, with which one of the authors is affiliated, approved the research methods (IRB No. 2107/002-010).

The survey was conducted using the consumer panel of an online survey company in South Korea, employing a two-between-subject design. The participants were randomized to a treatment group based on the body sizes of the stimuli (thin-size vs. plus-size). First, an email invitation was sent to panelists, informing them of the study. If they decided to participate, the email provided them with the URL to access the survey. Before starting the survey, all participants received detailed information about the purpose of this study, procedures, duration, potential advantages and drawbacks of participation, privacy protections, and compensation for their involvement. After fully understanding the study details, they electronically consented to participate. Participants were randomly assigned to one of the two stimulus groups. After viewing the stimuli for at least 15 seconds, they answered the questionnaire items. The participants were compensated modestly through the online survey company.

The collected data were analyzed through descriptive analysis, independent sample *t*-tests, confirmatory factor analysis (CFA), ANCOVA, and multi-group structural equation modeling (SEM). Additionally, the participants’ BMI was included as a covariate in the ANCOVA and multi-group SEM to eliminate the influence of participants’ BMI on the hypothesized relationships.

### Instrumentation

Questionnaire items were developed on a 5-point Likert scale (1 = “*strongly disagree”* and 5 = “*strongly agree”*), drawing on prior research. Perceived familiarity was assessed using four items from the study of Lei and Kim [34], with a Cronbach’s *α* of 0.76 in their study. Perceived physical attractiveness was measured using three items from Ahearne et al.’s study, with a Cronbach’s *α* of 0.76 in the study [59]. Six items from Borau and Bonnefon’s study were used to measure actual and ideal self-congruence [10], with their reliabilities confirmed through Cronbach’s *α* values (0.91 and 0.97) in their study. Lastly, purchase intention was assessed using two items from Spears and Singh [60], with a composite *α* of 0.97. These items were translated into Korean in prior studies through back-translation for data collection from South Korean participants: (a) perceived physical attractiveness and perceived familiarity from Lei and Kim’s [34] and Kim et al.’s [22] studies; (b) actual self-congruence and ideal self-congruence from Kim’s study [61]; and (c) purchase intention from Kim and Jung’s study [62].

## Results

### Measurement model results

We estimated the CFA with the collected data using AMOS and SPSS statistics programs, and the results indicated a satisfactory model fit (*X*^*2*^ = 230.425, *df* = 80, *X*^*2*^*/df* = 2.880, *p* = .000, NFI = .972, RFI = .963, IFI = .981, TLI = .975, CFI = .981, RMSEA = .055), as displayed in Table 1. Reliability measures were confirmed, with each Cronbach’s *α* coefficient exceeding 0.7 (ranging from .884 to .969). Convergent validity was established using Fornell and Larcher’s criteria [63]: (a) all standardized factor loadings were above 0.5 (ranging from .789 to .977), (b) all AVEs exceeded 0.5 (ranging from .695 to .915), and (c) all CRs were above 0.7 (ranging from .887 to .970). Discriminant validity was also confirmed based on Fornell and Larcher’s criteria [63], as displayed in Tables 1 and 2.

**Table 1:** Results of the confirmatory factor analysis.

Variables	Items	Std. Loading	*t*-value	AVE	CR	Cronbach’s α
**Familiarity**	FA1: I am familiar with the model in this ad.	.862	27.338	.695	.901	.900
	FA2: I feel comfortable with the model in this ad.	.800	24.315			
	FA3: The model in this ad is not new to me.	.789	24.247			
	FA4: I feel a sense of intimacy with the model in this ad.	.871	-			
**Physical Attractiveness**	PA1: The model in this ad is very good-looking.	.796	23.187	.724	.887	.884
	PA2: The model in this ad has an attractive appearance.	.902	26.458			
	PA3: The model in this ad is attractive.	.851	-			
**Actual Self- Congruence**	CAS1: The physical appearance of the model in the ad matches my physical appearance.	.934	-	.915	.970	.969
	CAS2: My physical appearance is similar to the model in the ad.	.977	29.315			
	CAS3: I physically resemble the model in the ad.	.958	28.238			
**Ideal Self- Congruence**	CIS1: The physical appearance of the model in the ad fits the physical appearance I would like to have.	.841	50.246	.782	.915	.913
	CIS2: I would like my physical appearance to be similar to the model in the ad.	.919	62.954			
	CIS3: I would like to physically resemble the model in the ad.	.891	-			
**Purchase** **Intention**	PI1: I have a strong interest in buying the product in this ad.	.926	28.205	.863	.926	.926
	PI2: I want to buy the product in this ad.	.932	-			
**Model Fit** **Indices**	*X*^2^ = 230.425, *df* = 80, *X*^*2*^*/df* = 2.880, *p* = .000, NFI = .972, RFI = .963, IFI = .981, TLI = .975, CFI = .981, RMSEA = .055					

N = 623

**Table 2:** Inter-construct correlation estimates.

Variance	FA	PA	ASC	ISC	PI
**FA**	.695^a^				
**PA**	.017^b^	.724			
**ASC**	.011	.006	.915		
**ISC**	.027	.068	.082	.782	
**PI**	.114	.056	.042	.054	.863

a: Average variance extracted (AVE) for constructs are displayed on the diagonal.

b: Numbers below the diagonal are squared correlation estimates of two variables.

PA = physical attractiveness, FA = familiarity, ASC = actual self-congruence, ISC = ideal self-congruence, PI = purchase intention

### SEM analysis of the S-O-R model

Following the validation of validity and reliability, we applied SEM using participants’ BMI as a covariate variable for testing hypotheses from H1 to H4. The results indicated a good model fit (*X*^*2*^ = 284.775, *df* = 91, *X*^*2*^*/df* = 3.129, *p* = .000, NFI = .965, RFI = .954, IFI = .976, TLI = .968, CFI = .976, RMSEA = .059), as outlined in Table 3 [64]. The proposed hypotheses, with the exception of the relationships from actual self-congruence to perceived physical attractiveness (H3a) and from ideal self-congruence to perceived familiarity (H4b), were found to be significant. Specifically, actual self-congruence (H1a: *β* = .167, *p* < .01) and ideal self-congruence (H1b: *β* = .112, *p* < .05) were favorably related to purchase intention towards the advertised products. The influences of perceived physical attractiveness (H2a: *β* = .244, *p* < .01) and perceived familiarity (H2b: *β* = .450, *p* < .01) on purchase intention were also significant. The relationship between actual self-congruence and perceived physical attractiveness was significantly negative (H3a: *β* = -.117, *p* < .01), leading to the rejection of H3a. In contrast, the influence of actual self-congruence on perceived familiarity was significant (H3b: *β* = .177, *p* < .01). The hypothesized relationship from ideal self-congruence to perceived physical attractiveness was supported (H4a: *β* = .550, *p* < .01), whereas the relationship from ideal self-congruence to perceived familiarity was rejected (H4b: *β* = .029). Regarding the influence of BMI as a covariate, the influence on perceived physical appearance was marginally significant (*β* = -.071, *p* < .10), and that on perceived familiarity was insignificant (*β* = .000). Lastly, the influence of the covariate on purchase intention was significant (*β* = .072, *p* < .05). Therefore, hypotheses H1a-H4b were accepted, except for H3a and H4b (refer to Table 3).

**Table 3:** Results of the SEM analysis for the S-O-R model.

Structural Path		All Participants (N = 623)			
		Std. Estimate	S. E.	CR	*p*
H1a	ASC → PI	.167	.047	4.034	.000
H1b	ISC → PI	.112	.050	2.306	.021
H2a	PA → PI	.244	.051	5.701	.000
H2b	FA → PI	.450	.041	11.843	.000
H3a	ASC → PA	**-.117**	**.043**	**-2.561**	**.010**
H3b	ASC → FA	.177	.053	3.549	.000
H4a	ISC → PA	.550	.044	10.886	.000
H4b	ISC → FA	**.029**	**.049**	**.576**	**.564**
Cova-riate	BMI → PA	-.071	.007	-1.184	.060
	BMI → FA	.000	.009	-.003	.997
	BMI → PI	.072	.008	2.134	.033
Model Fit Indices		*X*^2^ = 284.775, *df* = 91, *X*^*2*^*/df* = 3.129, *p* = .000, NFI = .965, RFI = .954, IFI = .976, TLI = .968, CFI = .976, RMSEA = .059			

PA = physical attractiveness, FA = familiarity, ASC = actual self-congruence, ISC = ideal self-congruence, PI = purchase intention

### ANCOVA results on perceptions of research variables

ANCOVA was employed to examine differences in the S-O-R framework between the two groups, for testing H5, with participants’ BMI used as a covariate (see Table 4). A significant difference was observed in the perceptions of ideal self-congruence between the two model types (H5b: *f(1*,*620)* = 117.908, *p* < .01), but not in actual self-congruence (H5a: *f(1*,*620)* = 1.034). Participants perceived higher ideal self-congruence with the thin-size model than with the plus-size model (H5b: *M*_thin-size_ = 2.698, *SD*_thin-size_ = .856; *M*_plus-size_ = 2.000, *SD*_plus-size_ = .751). With respect to perceived attractiveness, physical attractiveness (H5c: *f(1*,*620)* = 20.898, *p* < .01) and familiarity (H5d: *f(1*,*620)* = 78.344, *p* < .01) significantly varied for the two groups. The thin-size model was perceived as more physically attractive (H5c: *M*_thin-size_ = 3.513, *SD*_thin-size_ = .706; *M*_plus-size_ = 3.329, *SD*_plus-size_ = .775) but less familiar (H5d: *M*_thin-size_ = 2.905, *SD*_thin-size_ = .744; *M*_plus-size_ = 3.420, *SD*_plus-size_ = .706) than the plus-size model. Purchase intention towards the advertised products turned out to be significantly different between the two groups (H5e: *f(1*,*620)* = 34.493, *p* < .01). Participants exposed to the thin-size fashion model perceived a statistically higher intention to buy the advertised products than those exposed to the plus-size model (H5e: *M*_thin-size_ = 3.049, *SD*_thin-size_ = .887; *M*_plus-size_ = 2.649, *SD*_plus-size_ = .798). The covariate’s influence was not significant across all H5 hypotheses. Therefore, all H5 hypotheses were supported except for H5a.

**Table 4:** Results of ANCOVA by the sizes.

Hypothesis	Variable	Group	Mean	S.D.	Group Difference *f*-value *(df)*	BMI (Covariate) *f*-value *(df)*
H5(a)	ASC	Thin-Size	1.942	.715	1.034 (1, 620)	1.647 (1, 620)
		Plus-Size	1.884	.775		
H5(b)	ISC	Thin-Size	2.698	.856	117.908*** (1, 620)	1.603 (1, 620)
		Plus-Size	2.000	.751		
H5(c)	PA	Thin-Size	3.513	.706	20.898*** (1, 620)	1.535 (1, 620)
		Plus-Size	3.239	.775		
H5(d)	FA	Thin-Size	2.905	.744	78.344*** (1, 620)	.000 (1, 620)
		Plus-Size	3.420	.706		
H5(e)	PI	Thin-Size	3.049	.887	34.493*** (1, 620)	2.657 (1, 620)
		Plus-Size	2.649	.798		

***p < .01

PA = physical attractiveness, FA = familiarity, ASC = actual self-congruence, ISC = ideal self-congruence, PI = purchase intention

### Moderation effects of fashion model’s size

To verify factorial invariance, we imposed an equality constraint on the measurement weights, employing Drasgow and Kanfer’s method [65]. The participants’ BMI was used as a covariate in calculating the chi-square difference between the baseline unconstrained model and the constrained model that evaluated factor loading invariance across the two groups exposed to the thin-size vs. plus-size fashion model stimuli. The analysis showed that the chi-square difference was not statistically significant (*ΔX*^*2*^(*10*) = 16.919, *p* > .05). Consequently, we confirmed that the measurement items were suitable for the multi-group analysis. After verifying the model fit indices of the unconstrained model (*X*^*2*^ = 358.424, *df* = 182, *X*^*2*^*/df* = 1.969, *p* = .000, NFI = .956, RFI = .942, IFI = .978, TLI = .970, CFI = .978, RMSEA = .040), we tested the hypotheses from H6 to H9.

Actual self-congruence (H6a: *ΔX*^*2*^ = .109, *Δdf* = 1) and ideal self-congruence (H6b: *ΔX*^*2*^ = 1.282, *Δdf* = 1) did not have statistically different influences on purchase intention between the two groups. Thus, H6a and H6b were rejected. The difference in the effect of perceived physical attractiveness on purchase intention of advertised products was marginally significant between the two groups (H7a: *ΔX*^*2*^ = 2.712, *Δdf* = 1, *p* < .10), but we interpreted this result as significant due to the increased tendency to accept marginally significant effects as proof of a difference [66]. Nevertheless, we rejected H7a since the influence in the group exposed to the plus-size model was greater than that in the thin-size group, which contradicted H7a (i.e., *β*_H2a:thin-size_ > *β*_H2a:plus-size_). The influence of perceived familiarity on purchase intention was still significant and positive in both groups, but the difference in the relationship was not significant (H7b: *ΔX*^*2*^ = .575, *Δdf* = 1). Thus, H7b was not supported.

There was no statistically significant difference between the two groups in terms of the influence of actual self-congruence on perceived physical attractiveness (H8a: *ΔX*^*2*^ = .404, *Δdf* = 1); thus, H8a was rejected. However, the impact of actual self-congruence on perceived familiarity was significantly positive for those exposed to the thin-size model (H8b_thin-size:_ *β* = .146, *p* < .05), but not significant for those exposed to the plus-size model (H8b_plus-size:_ *β* = -.048, *p* = .572). Thus, H8b was supported given that the influence in the thin-size model group was significantly greater than that in the plus-size model group (H8b: *ΔX*^*2*^ = 3.962, *Δdf* = 1, *p* < .05).

Ideal self-congruence demonstrated a statistically distinct influence on perceived physical attractiveness between the two groups (H9a: *ΔX*^*2*^ = 7.157, *Δdf* = 1, *p* < .01). However, H9b was rejected because the influence in the plus-size model group (H9a_plus-size:_ *β* = .674, *p* < .01) was stronger than the influence in the thin-size model group (H9a_thin-size:_ *β* = .496, *p* < .01), which was opposite of the expected direction. The influence of ideal self-congruence on perceived familiarity did not differ significantly between the two groups (H9b: *ΔX*^*2*^ = .000, *Δdf* = 1). Thus, H9b was rejected. Table 5 describes the findings of the multi-group analysis.

**Table 5:** Results of the multi-group comparison analysis (Covariate = BMI).

Structural Path		Thin-Size Model Group (n = 316)				Plus-Size Model Group (n = 307)				Comparison (*Δdf = 1)*	
		Std. Estimate	S. E.	CR	*p*	Std. Estimate	S. E.	CR	*p*	*ΔX* ^*2*^	Sig.
H6a	ASC → PI	.135	.061	2.406	.016	.101	.078	1.451	.147	0.109	NS
H6b	ISC → PI	.321	.069	4.837	.000	.163	.104	1.875	.061	1.282	NS
H7a	**PA → PI**	**.181**	**.067**	**3.214**	**.001**	**.328**	**.073**	**5.057**	**.000**	**2.712**	***P* < .10**
H7b	FA → PI	.309	.057	5.709	.000	.329	.065	6.017	.000	0.575	NS
H8a	ASC → PA	-.178	.057	-2.813	.005	-.224	.078	-2.865	.004	0.404	NS
H8b	**ASC → FA**	**.146**	**.067**	**2.242**	**.025**	**-.048**	**.080**	**-.566**	**.572**	**3.962**	***P* < .05**
H9a	**ISC → PA**	**.496**	**.060**	**7.192**	**.000**	**.674**	**.092**	**7.842**	**.000**	**7.157**	***P* < .01**
H9b	ISC → FA	.294	.066	4.402	.000	.287	.089	3.254	.001	0.000	NS
Cova-riate	BMI → PA	-.177	.010	-3.209	.001	-.010	.011	-.179	.858	4.263	*P* < .05
	BMI → FA	-.044	.012	-.775	.438	.049	.011	.797	.426	1.277	NS
	BMI → PI	.022	.011	.450	.653	.102	.011	2.108	.035	1.328	NS
Model Fit Indices		*X*^2^ = 358.424, *df* = 182, *X*^*2*^*/df* = 1.969, *p* = .000, NFI = .956, RFI = .942, IFI = .978, TLI = .970, CFI = .978, RMSEA = .040									

## Discussion and conclusion

### Theoretical contributions

The West has seen a rise in inclusive marketing efforts targeting various marginalized groups [67], yet South Korean domestic fashion brands have not fully embraced such marketing practices, including the body positivity movement [5]. This hesitancy may stem from the challenge of creating inclusive advertisements that represent societal minorities, such as South Koreans with obesity. While these advertisements could yield positive societal impacts, they may also pose risks to brand profitability [52]. Notably, previous studies examining the effects of inclusive advertising from non-Western perspectives are scarce [68]. Therefore, the current study illuminates the potential for adopting inclusive advertising strategies in Asian markets, particularly through the integration of plus-size representation.

In the Asian context, the anticipated positive effects of self-congruence on the perceived attractiveness of a fashion model, regardless of the model’s body size, were not fully supported. Specifically, we found distinct roles for actual and ideal self-congruence. Participants experienced increased perceived familiarity when they viewed a model as congruent with their actual selves, but this congruence negatively impacted perceived physical attractiveness. Conversely, a model congruent with the participants’ ideal selves was deemed physically attractive but not familiar. Importantly, purchase intentions were positively influenced when participants perceived an advertising model as congruent with their self-concept, covering both actual and ideal self-congruence.

The negative response of South Korean women to fashion items advertised by a plus-size model was notable. Given the prevailing thin body ideals and the deep-rooted preference for slimness among Korean women [12, 53, 58], a thin-size model was perceived as embodying the stereotypical and successful majority, resulting in higher physical attractiveness and greater congruence with the participants’ ideal selves. Thus, they were inclined to purchase items modeled by the thin-size model. Interestingly, even though the participants’ physical measurements matched that of the thin-size model, they did not perceive themselves as resembling either the thin-size or the plus-size model. Given that the participants viewed their ordinary or even thin bodies as larger than they actually were [69], it is reasonable to infer that South Korean women perceive their body sizes as bigger than that of the thin-size model yet slimmer than that of the plus-size model. These findings align with previous research indicating that South Korean women have lower levels of appearance-related self-esteem [53] and tend to perceive their bodies as larger than their actual sizes [69]. Therefore, it appears that South Korean women aspire to unachievable beauty ideals that are impracticable in reality [53].

Perceived familiarity was the only dimension where South Korean female consumers exhibited a higher perception of the plus-size model compared to the thin-size model. This outcome suggests a new direction for the impact of plus-size model advertising in the South Korean fashion industry, diverging from recent studies reporting negative responses to advertisements featuring plus-size models in South Korea [5, 14]. Future studies could refine the construct of perceived familiarity into “ordinary familiarity,” as suggested by Hwang and Kim [70], who proposed two dimensions of endorser familiarity: multiple endorser familiarity and ordinary familiarity. Traditionally, recurrent exposure to endorsers within formal advertising settings cultivates multiple endorser familiarity, the predominant conceptualization within advertising scholarship [70]. Given the prevalence of global advertisements featuring plus-size models in the South Korean fashion market [5], frequent media exposure could enhance perceived familiarity with multiple endorsers, including plus-size models. However, thin-size models have traditionally been more dominant in the South Korean fashion industry doe to longer exposure durations and higher frequency in advertisements [5]. Thus, this study emphasizes “ordinary familiarity,” suggesting that intimacy and familiarity may cause consumers to perceive endorsers as ordinary, nearby, and next-door individuals [70]. In South Korean, the term “familiar” is often used to describe average or plus-size body shapes, contrasting with the slim physiques typically associated with celebrities [71]. Consequently, South Korean female participants, particularly those in their 20s and 30s, found the plus-size model more familiar than the thin-size model (i.e., reflecting celebrities’ or conventional models’ shapes), even though they believed the plus-size shape did not match their own body sizes.

In light of potential concerns regarding the effects of inclusive advertising, we hypothesized that the relationships between both actual and ideal self-congruence and purchase intention, as well as between perceived familiarity and purchase intention, would be greater in the consumer group exposed to the thin-size model than the other group exposed to the plus-size model. However, regardless of the model types exposed to the participants, these relationships remained statistically indistinguishable, contrary to our expectations. On the other hand, the perceived physical attractiveness of the plus-size model demonstrated an unexpectedly positive influence on purchase intention, surpassing that of the thin-size model. When South Korean female consumers perceived congruence between the plus-size model and their ideal selves, the influence of ideal self-congruence on the plus-size model’s perceived physical attractiveness was more pronounced than that in the group involving the thin-size model. These findings imply that South Korean female consumers may be more disposed to find plus-size models physically appealing when they perceive the models as aligning with their ideal selves, thus boosting their intent to purchase products modeled by the plus-size figures. Despite prior studies that employing plus-size models in South Korean fashion advertising might lack economic viability [5, 14], our findings indicate that advertisements featuring physically attractive plus-size models can indeed elicit favorable reactions from South Korean consumers.

Additionally, this study explored the effects of perceived actual self-congruence on the perception of advertising models, particularly regarding perceived physical attractiveness and familiarity. South Korean women who deemed their actual body sizes congruent with a model’s tended to view the model as physically unattractive, irrespective of the model’s body size. In terms of perceived familiarity, exposure to thin-size models, as opposed to plus-size models, heightened the perception of congruence with the participants’ actual selves, thereby increasing perceived familiarity. These findings suggested that lower level of self-esteem levels concerning South Korean women’s body sizes may decrease the potential positive impact of actual self-congruence on endorsers in fashion advertising (78).

### Managerial contributions

This study’s insights offer several managerial implications for fashion apparel advertisers formulating marketing strategies in Asian markets. First, as size-inclusive advertisements become more familiar within Asian cultures, they may present additional opportunities for favorable outcomes. Marketers targeting Asian consumers should cultivate perceived familiarity through repeated exposure to inclusive advertisements featuring ordinary people or plus-sized celebrities, moving beyond traditional thin and slender standards. Second, advertisements should emphasize ideal self-congruence, even when featuring plus-size models. Asian women often underestimate their body shapes (69) and pursue ideal beauty [53]. Hence, they are drawn to endorsers and fashion products that resonate with their ideal selves. However, with the global spread of body positivity, even Asian women adhering to stringent thin body standards are beginning to recognize a healthy and muscular body as the new ideal [5]. Thus, the key to successful inclusive advertising lies not in accurately reflecting the real-life body shapes prevalent in the Asian market but in crafting an idealized advertising portrayal of plus-size body shapes. Lastly, marketers should incorporate inclusivity in terms of body sizes and shapes, traditionally based on Western size ranges. Global fashion advertisements can enhance inclusivity by featuring Asian plus-size models with varying heights, body shapes, and sizes, thereby increasing perceived familiarity among Asian consumers. In size-inclusive advertisements depicting models with diverse body sizes, it is essential to highlight the attractive qualities of each body size distinctly.

### Limitations and future research

Despite its theoretical and practical contributions, this study faces several limitations that warrant further exploration. First, the influence of key variables in our research framework may differ across various contexts, such as the advertising medium (e.g., social media, offline retail), the nature of the models (e.g., celebrity, non-celebrity), the models’ ethnic backgrounds (e.g., Asian, Caucasian, African American), targeted demographic segments (e.g., adolescents, elderly consumers), and the category of apparel products (e.g., sportswear, luxury goods, casual wear). Future research should address the multifaceted nature of inclusive fashion advertising within these diverse settings.

Second, the research framework did not include consumer traits that could potentially influence the examined relationships. Future inquiries are encouraged to incorporate variables such as personality, appearance-related self-esteem, involvement in fashion products, and the perception of body positivity. The exploration of lower self-esteem levels among Asian female consumers is particularly crucial [53, 56], given its potential to exacerbate negative reactions to size-inclusive advertising in Asian markets.

Third, the current research primarily focused on purchase intention towards the advertised products as the outcome variable. However, it is essential to acknowledge that such intentions might be shaped by the intrinsic attributes of the products, such as design, color, textiles, or individual consumer preferences, rather than solely by perceptions of the fashion models. Therefore, subsequent studies should develop frameworks that examine outcome variables directly influenced by the attributes of advertising models, including attributes towards the advertisement, the models, or the brands represented.

Last, considering this study’s focus on inclusive advertisements from global fashion brands of Western origin, employing Caucasian women as stimuli, the perception of Caucasian models’ body shapes by Asian female participants might diverge from perceptions of Asian models. Future investigations should include Asian models of various sizes to more accurately access Asian consumers’ reactions to such representations. Given these limitations, it is imperative for future research to expand the scope of research settings, outcome variables, consumer characteristics, and cultural representations in the study of inclusive fashion advertising.
